# Adding Cyclooxygenase Inhibitors to Immune Checkpoint Inhibitors Did Not Improve Outcomes in Metastatic Renal Cell Carcinoma

**DOI:** 10.3390/cells11162505

**Published:** 2022-08-12

**Authors:** Yumeng Zhang, Premsai Kumar, Jacob J. Adashek, William P. Skelton, Jiannong Li, Aram Vosoughi, Jad Chahoud, Brandon J. Manley, Philippe E. Spiess

**Affiliations:** 1 Division of Hematology and Medical Oncology, H. Lee Moffitt Cancer Center, University of South Florida, Tampa, FL 33612, USA; 2 Morsani College of Medicine, University of South Florida Health, Tampa, FL 33612, USA; 3 Department of Oncology, The Sidney Kimmel Comprehensive Cancer Center, The Johns Hopkins Hospital, Baltimore, MD 21218, USA; 4 Department of Genitourinary Oncology, University of Virginia, Charlottesville, VA 22903, USA; 5 Department of Biostatistics, H. Lee Moffitt Cancer Center & Research Institute, Tampa, FL 33612, USA; 6 Department of Anatomical Pathology, H. Lee Moffitt Cancer Center & Research Institute, Tampa, FL 33612, USA; 7 Department of Genitourinary Oncology, H. Lee Moffitt Cancer Center & Research Institute, Tampa, FL 33612, USA

**Keywords:** cyclooxygenase inhibition, NSAID, aspirin, immune checkpoint inhibitors, immunotherapy, renal cell carcinoma

## Abstract

Modulating the cyclooxygenase 2 (COX-2) pathway has improved responses to immune checkpoint inhibitors (ICIs) in certain solid tumors, such as melanoma. Little is known about COX-2 inhibition in response to ICIs in metastatic renal cell carcinoma (mRCC). In this retrospective cohort study, we examined the effect of COX-2 inhibitors on the long-term outcomes of mRCC patients undergoing ICI therapies. Among 211 patients with mRCC, 23 patients were excluded due to loss to follow-up. Among 188 included patients, 120 patients received either an NSAID or aspirin for at least three weeks during ICI therapies. Clear cell histology was present in 96% of cases. The median overall survival (OS) was similar regardless of the COX inhibitor (COXi) (i.e., NSAID or aspirin) use (27 months for COXi vs. 33 months for no-COXi groups; *p* = 0.73). The no-COXi group showed a trend toward longer median progression-free survival (8 months for COXi vs. 13 months for no-COXi groups; *p* = 0.13). When looking specifically at NSAID use in a multivariate analysis, NSAID use was associated with a higher risk of progression (HR = 1.52 [95% CI, 1.04–2.22]) and death (HR = 1.60 [95% CI, 1.02–2.52]). In summary, COXis did not improve disease control or survival among patients with mRCC who were undergoing ICI therapies. Instead, the concurrent use of NSAIDs was associated with worse outcomes. Larger studies are needed to validate our observation.

## 1. Introduction

Renal cell carcinoma (RCC) is one of the fastest-growing malignancies worldwide and has more than doubled its incidence in the past decades [1]. It is the ninth most common malignancy and accounts for more than 13,000 deaths in the United States annually [1,2]. Since 2015, immune checkpoint inhibitors (ICIs) have changed the therapeutic landscape of RCC [3]. In 2018, a dual immune checkpoint blockade with ipilimumab and nivolumab was the first ICI-based therapy approved in the frontline setting by the FDA (for intermediate- and poor-risk metastatic clear cell RCC (mccRCC)). Subsequently, several ICI (pembrolizumab, nivolumab, and avelumab) and tyrosine kinase inhibitors (TKI) (axitinib, lenvatinib, and cabozantinib) combinations have been approved as frontline therapies for mccRCC. Though the initial response rates for dual ICI blockade and ICI+TKI combinations are high, responses are usually not sustained for many patients [4]. Many ongoing studies have attempted to extend the responses to ICI therapies through immunomodulation.

The cyclooxygenase 2 (COX-2) pathway plays an essential role in inflammation, tumor growth, metastasis, and angiogenesis. Murine models show that COX-2–mediated prostaglandin E production could exert immunomodulatory effects on dendritic cells, monocyte-derived macrophages, and regulatory T cells, thereby promoting immune escape [5]. COX inhibition with aspirin and anti–PD-1 monoclonal antibodies could induce rapid tumor regression [5]. In retrospective clinical studies, the combined COX blockade and ICIs improved the objective response rate and time to progression among patients with metastatic melanoma and NSCLC [6]. COX-2 inhibition can potentially negate the effects of a high neutrophil-to-lymphocyte ratio (NLR) [7] and differential effects based on PD-L1 expression [8] in melanoma. Ongoing clinical trials are evaluating combination therapies with COX-2 inhibitors and PD-1 inhibitors in colorectal cancer (NCT03026140 and NCT03926338) and breast cancer (NCT04188119 and NCT04348747) [7].

Little is known about the effects of COX-2 inhibition on modulating responses to ICIs in metastatic renal cell carcinoma (mRCC). We conducted a retrospective cohort study to determine whether concurrently using COX inhibitors (COXi) with ICI therapy would result in longer disease control and improved survival outcomes among patients with mRCC.

## 2. Materials and Methods

### 2.1. Patient Population

We retrospectively reviewed patients with mRCC who initiated their first course of immunotherapy (can be as the first line or subsequent lines) in the metastatic setting between June 2014 and July 2019 at the Moffitt Cancer Center. Patients with concurrent diagnoses of other malignancies were excluded, except for non-melanomatous skin cancer or localized prostate cancer. The immunotherapy could either be a single agent (e.g., pembrolizumab, nivolumab, durvalumab, atezolizumab), a dual agent (e.g., nivolumab/ipilimumab), or in combination with TKIs or other investigative agents in a clinical trial (e.g., interleukins). COXi use was defined as using a COXi during the immunotherapy for at least 3 weeks. COXis included NSAIDs or aspirin.

### 2.2. Study Measurement

Baseline clinical and treatment data were extracted from electronic medical records and included sex; race; age at diagnosis; age at immunotherapy treatment; vital status; date of last visit/death; the Karnofsky Performance Scale score; histology subtype; and an International Metastatic RCC Database Consortium (IMDC) risk score and pretreatment NLR from peripheral blood within 1 week of starting ICI. Disease progression was determined by the treating physician using imaging (CT, PET, or MRI) as documented in the medical records.

### 2.3. Statistical Analyses

Patients were stratified based on COXi use. The baseline clinical characteristics were summarized using descriptive statistics. The median and interquartile ranges were used for continuous variables and proportions and frequencies were used for categorical variables. To compare differences among the groups, Kruskal-Wallis tests were conducted for continuous variables and Chi-squared tests were conducted for categorical variables. All *P* values were 2-sided.

For survival analyses, the overall survival (OS) of all patients was defined as the time of the immunotherapy initiation to the date of death or censorship at the last date known alive. Progression-free survival (PFS) was calculated from the time of the immunotherapy initiation to the date of disease progression or otherwise censored at the last date known alive. The Kaplan-Meier method was used for OS, and PFS analysis and log-rank tests were adopted to compare survival differences between the groups. A univariate Cox proportional hazards (PH) model was constructed to evaluate the association of OS and PFS with the individual clinical features, including sex; age at diagnosis; age at treatment; type of therapies; histology subtype (clear cell vs. non-clear cell); IMDC risk score; lines of prior therapies; and NLR. Multivariate Cox proportional hazard models were constructed to further evaluate the association of OS and PFS with clinical features selected by backward stepwise model selection based on the Akaike Information Criterion using the MASS R package. All statistical analyses were performed using the R 4.2.0 software (https://www.R-project.org (accessed on 20 June 2022)).

## 3. Results

### 3.1. Demographics and Clinical Characteristics

**Figure 1:**
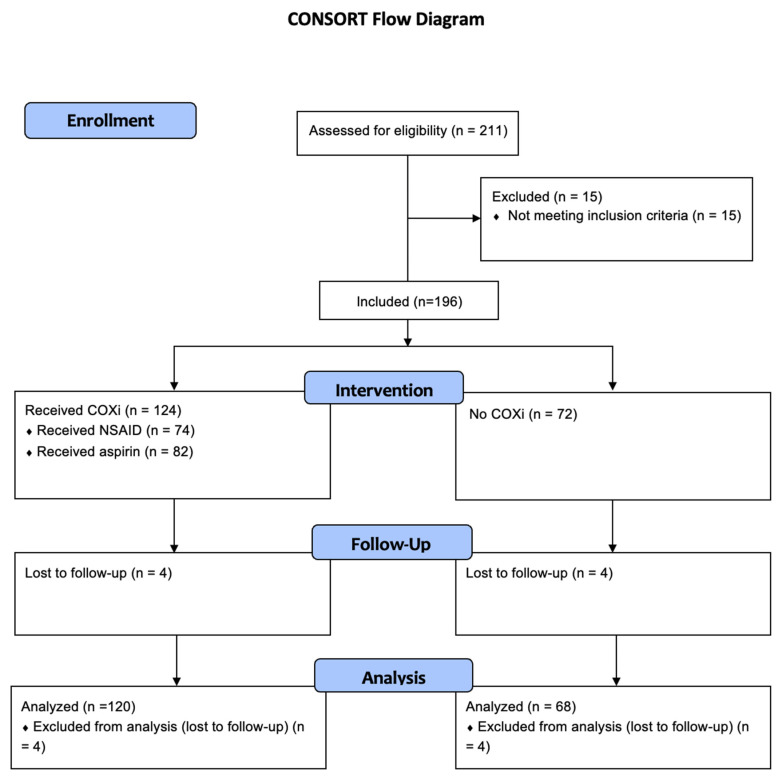
CONSORT flow diagram of patient distribution.

A total of 211 patients who received ICI therapy from 1 January 2014 to 31 December 2019 at the Moffitt Cancer Center were identified. Among them, 188 patients met the inclusion criteria and 23 of these patients were excluded from the analysis: 8 patients because of lack of follow-up information after the first cycle of therapy and 15 patients because of concurrent diagnosis of other malignancies (Figure 1).

Most patients were white males, and most had clear cell RCC as their histology (Table 1). A total of 154 patients (82%) had nephrectomy prior to the initiation of ICI. A total of 120 patients received either NSAIDs or aspirin. Among these patients, 82 received aspirin and 70 received NSAIDs. Thirty-two patients received both NSAIDs and aspirin. Regarding aspirin dosing, all patients received 81 mg daily except for three patients who received 325 mg daily. Among 70 patients who received NSAIDs, 66 patients received non-selective COX inhibitors, e.g., naproxen, ibuprofen, meloxicam, diclofenac; while four patients received a selective COX inhibitor, celecoxib. 

For patients who received NSAIDs or aspirin, the median age at treatment was 66 years (interquartile range, 59–71 years), which was higher than that of patients who did not receive either NSAIDs or aspirin (interquartile range, 51–71 years) (*p* = 0.011) (Table 1). When examining NSAID and aspirin use separately, the age difference was primarily attributed to the aspirin-use group. Other clinicopathological characteristics were similar between the two groups.

Of the total included patients, 56% were treated with PD-1/PD-L1 inhibitor monotherapy; 21% with a dual blockade therapy; and 6% with PD-1 inhibitor and TKI combination therapy. The remaining 17% of patients were treated under a clinical trial with immunotherapy combined with interleukins. The distribution of immunotherapy types was similar between the two groups (Table 1).

### 3.2. OS Analysis by Treatment Received

**Figure 2:**
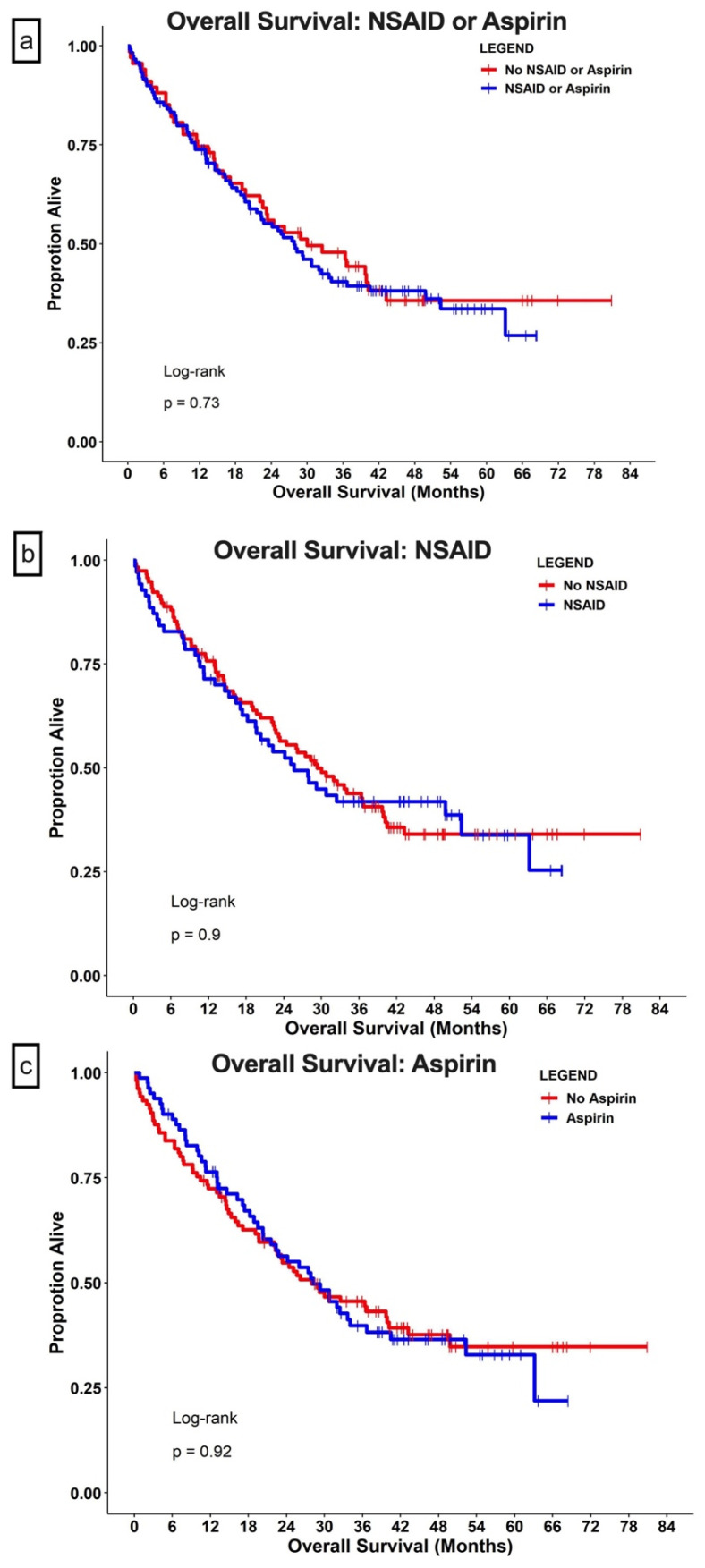
**Overall survival.** Patients with metastatic renal cell carcinoma were grouped based on concurrent use of (**a**) COX inhibitors (aspirin or NSAID); (**b**) use of aspirin; or (**c**) use of NSAIDs. The dots in all panels indicate censored data.

At the end of the study, 113 (60%) patients were deceased. The median follow-up time was 24.6 months (95% CI, 18.2–30.7 months) for the COXi group (i.e., the use of either NSAIDs or aspirin) and 27.3 months (95% CI, 17.1–36.5 months) for the no-COXi group. The median OS was similar for patients with concurrent COXi (27 months for the COXi group vs. 33 months for the no-COXi group; HR = 1.09; 95% CI, 0.75–1.6; *p* = 0.65) (Figure 2a). When evaluating NSAID use and aspirin use separately using KM methods, the median OS did not differ significantly between the NSAID use group vs the no-NSAID-use group (i.e., all patients who did not receive NSAID) and the aspirin-use group vs the no-aspirin-use group (i.e., all patients who did not receive aspirin) (Figure 2b,c).

In univariate analyses, neither NSAID nor aspirin use demonstrated an impact on the OS (Table 2). Non-clear-cell histology, poor IMDC risk group classification, more lines of prior therapies, and high NLR were associated with shorter survival (Table 2); ICIs in combination with interleukins in a clinical trial setting were associated with longer survival (HR = 0.19; 95% CI, 0.09–0.41; *p* < 0.001). Sex, age at diagnosis, and age at treatment did not impact survival. After adjusting for the type of ICI, histology subtype, IMDC risk group, prior lines of therapies, and NLR, NSAID use was associated with poorer survival, with an HR of 1.60 (95% CI, 1.02–2.52; *p* = 0.04). In a similar analysis, aspirin use did not impact survival (*p* = 0.89) (Table 2).

### 3.3. PFS Analysis by Treatment Received

**Figure 3:**
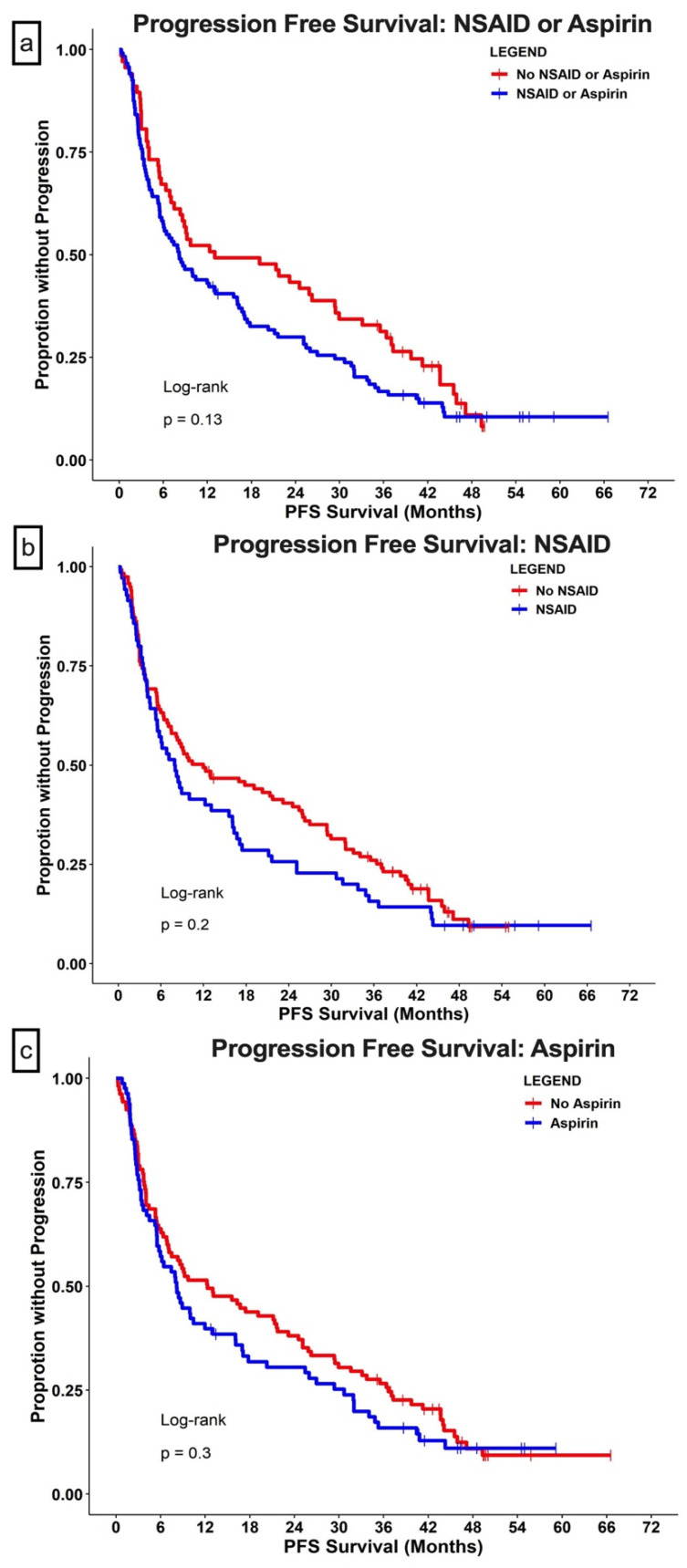
**Progression-free survival (PFS).** Patients with metastatic renal cell carcinoma were grouped based on concurrent use of (**a**) COX inhibitors (aspirin or NSAID); (**b**) use of aspirin; or (**c**) use of NSAID. The dots in all panels indicate censored data.

Though not statistically significant, the median PFS was shorter in the group with concurrent COX inhibition (8 months for the COXi group vs. 13 months for the no-COXi group; HR = 1.4; 95% CI, 0.98–1.9; *p* = 0.055) (Figure 3a). The 2-year PFS rate was higher in the no-COXi group (42% [95% CI, 30–52%]) than in the no-COXi group (28% [95% CI 21–37%]). When examining the types of COXis separately, both NSAID and aspirin use showed a trend toward shorter PFS (Figure 3b,c).

Poor IMDC risk group classification, more lines of prior therapies, and high NLR were associated with a higher likelihood of progression (Table 3). ICIs combined with interleukins in clinical trial settings were associated with longer PFS (HR = 0.38; 95% CI, 0.23–0.61; *p* < 0.001) compared to the PD-1 inhibitor group. NSAID use was associated with a higher likelihood of progression (HR = 1.52; 95% CI, 1.04–2.22; *p* = 0.03) in the multivariate analysis (Table 3).

## 4. Discussion

Our study demonstrated that combining COXis with ICIs did not result in longer disease control nor improved survival outcomes among patients with mRCC. Furthermore, NSAID use was associated with a higher probability of death (HR = 1.6) and disease progression (HR = 1.5) independent of IMDC risk group, age at treatment, pretreatment NLR, type of ICI received, and lines of prior therapies. As most of the included patients used non-selective COX inhibitors, the conclusion might not be applicable to selective COX inhibitors such as celecoxib.

There has been controversy over the effect of COX pathway inhibition on the response to ICIs for solid tumors. Wang et al. reported that concurrently using COXis and ICIs further delayed disease progression in metastatic melanoma and NSCLC than ICIs alone [6]. This effect is potentially mediated by reversing the negative prognostic effect of high NLR [6]. On the contrary, a retrospective study of 330 patients with metastatic melanoma did not find an association between NSAID use and improved outcomes [9]. Similarly, Sieber et al. showed similar rates of clinical benefits regardless of aspirin use among patients receiving ICI therapy for solid tumors in a meta-analysis [10].

To the best of our knowledge, no clinical studies have evaluated the effects of COX inhibition on metastatic RCC response to ICIs. Based on our data, not only did concurrent COX inhibition not improve the outcome of ICIs in mRCC but NSAID use was specifically associated with shorter PFS and OS. The explanations for the discrepancy might be twofold. First, the immune microenvironment might be intrinsically different between RCC, melanoma, and NSCLC. Though RCC has a high burden of tumor-infiltrating lymphocytes, unlike melanoma and NSCLC, ICIs rarely produce long-lasting responses for mRCC [11]. The reason for such differences remains unclear and is possibly related to the different immune microenvironments in the organ [11]. The mutation burden in a typical ccRCC is much less than that in melanoma or NSCLC, suggesting that RCC cells have limited expression of tumor-specific antigens [12]. In addition, the high tumor-infiltrating lymphocytes had a high-level expression of CTLA4 and PD-1, predicting a worse outcome in patients with ccRCC. This finding suggests that the cytotoxic function of T cells was limited by other suppressive immune cells in the environment [13]. Second, there might be a direct interaction between NSAIDs and RCC tumor biology. Though suppression of the COX-2 pathway using α-linolenic acid has inhibited RCC growth in in vitro studies [14], no benefits have been demonstrated in human studies. On the contrary, Bruinsma et al. reported that the use of NSAIDs is associated with an increased risk of developing RCC in women in a population-based case-control study [15]. The exact mechanism by which NSAID promotes RCC development is unknown. In addition, previous attempts failed to demonstrate improved response by adding COX-2 inhibitors to immunotherapies [16]. In an early phase 2 study examining interferon therapy for mRCC, adding celecoxib, a selective COX-2 inhibitor, did not improve response rate or time to progression [16].

This study had several limitations. First, though patients’ characteristics were similar between comparison arms, the retrospective nature of the study is subject to potential confounding biases from the unmeasured variables, such as medical comorbidities. Of note, CKD could potentially affect the use of NSAIDs. Unfortunately, eGFR was only available in 75 patients at the initiation of ICI. Among these patients, median eGFR was not significantly different between the NSAID group and the no NSAID groups. Among 25 patients in the NSAID group, 9 (36%) had an eGFR < 60, and 1 (4%) had an eGFR < 30. Among 50 patients in the no NSAID group, 21 (42%) patients had an eGFR < 60, and 6 (12%) had eGFR < 30. Second, our study included various lines of prior treatment and heterogeneous treatment-regimen combinations that included TKIs as well as interleukins in the setting of clinical trials. COXis can also potentially affect those therapies. However, the multivariate analyses did not show an interaction between treatment regimens, prior lines of therapies, and the use of COXis. Third, we did not analyze the individual histology subtypes. Due to the small number of patients that qualified for each subtype, we decided to combine the categories as non-clear-cell RCC.

Notwithstanding these limitations, the study elucidates the ongoing debate on whether COX inhibitors should be used among patients undergoing ICI therapies for metastatic malignancies. Further validation studies with larger cohorts are needed to confirm our findings.

## 5. Conclusions

In summary, the addition of COXis to ICI therapies did not improve disease control and survival among patients with mRCC. Instead, concurrent NSAID use was associated with a high probability of progression and death among patients with mRCC.

## Figures and Tables

**Table 1:** Characteristics of 188 Patients who Received Immune Checkpoint Inhibitors for Metastatic Renal Cell Carcinoma.

Group	COXi	No-COXi	*p* Value
**No. patients**	120	68	
**Sex, no. (%)**			0.99
Male	95 (79)	53 (78)	
Female	25 (21)	15 (22)	
**Age at diagnosis, median (IQR), years**	61 (55–67)	57 (48-68)	0.135
**Age at treatment, median (IQR), years**	66 (59–71)	60 (51–71)	0.011
**Race, no. (%)**			0.56
White	111 (94)	60 (90)	
Black	2 (2)	2 (3)	
Hispanic	4 (3)	3 (4)	
Asian	1 (1)	2 (3)	
**Type of ICI, no. (%)**			0.81
PD-1 inhibitors	70 (58)	35 (52)	
PD-1 inhibitors/CTLA4 inhibitors	24 (20)	16 (24)	
PD-1 inhibitors + TKI	7 (6)	4 (6)	
PD-1 + interleukins under clinical trials	19 (16)	13 (19)	
**Histology subtype, no. (%)**			1.0
Clear cell RCC	115 (96) *	65 (96)	
Non–clear-cell RCC	5 (4.1) *	3 (4.4)	
**IMDC risk ^^^, no. (%)**	111	60	0.112
Favorable	36 (32) *	11 (18) *	
Intermediate	59 (53) *	41 (68) *	
Poor risk	16 (14) *	8 (13) *	
**Lines of prior therapy, no. (%)**			0.503
0	45 (38)	31 (46)	
1	35 (29)	17 (25)	
2	24 (20)	9 (13)	
3 or more	16 (13)	11 (16)	
**NLR, no., median (95% CI) ^#^**	103, 3.2 (1.4–8.7)	57, 3.3 (1.2–11.4)	0.869

**Abbreviations**: COXi, cyclooxygenase inhibitor; CTLA4, cytotoxic T-lymphocyte-associated protein 4; ICIs, immune checkpoint inhibitors; IMDC, International Metastatic RCC Database Consortium Risk model; IQR, interquartile range; NLR, neutrophil-to-lymphocyte ratio; PD-1, programmed cell death protein 1; RCC, renal cell carcinoma; TKIs, tyrosine kinase inhibitors. * The percentages may not add up to 100 due to rounding. ^^^ Only 171 patients had available data at the time of metastatic disease to calculate the IMDC risk group. ^#^ Only 160 patients had available neutrophils and lymphocytes numbers during metastatic disease.

**Table 2:** Univariate and Multivariate Analysis of Factors Associated with Overall Survival.

Predictor	Univariate				Multivariate			
	HR	95% CI		*p* Value	HR	95% CI		*p* Value
**NSAID use**	1.03	0.70	1.5	0.90	1.60	1.02	2.52	0.04
**Aspirin use**	1.02	0.70	1.48	0.92	1.03	0.66	1.63	0.89
**Male**	0.85	0.55	1.32	0.47	—			
**Age at diagnosis**	1.00	0.99	1.02	0.75	—			
**Age at treatment**	1.00	0.99	1.02	0.63	1.01	0.99	1.03	0.46
**Type of ICI,**				<0.001				
PD-1 inhibitors	Reference				Reference			
PD-1 inhibitors/CTLA4 inhibitors	0.91	0.57	1.45	0.69	1.06	0.53	2.12	0.88
PD-1 inhibitors + TKI	1.00	0.48	2.09	0.99	1.81	0.77	4.24	0.17
PD-1 + interleukins under clinical trial	0.19	0.09	0.41	<0.001	0.16	0.06	0.43	<0.001
**Histology subtype**				0.0024				
Clear cell RCC	Reference				Reference			
Non-clear cell RCC	3.03	1.47	6.25	0.003	1.78	0.65	4.92	0.26
**IMDC risk**				<0.001				
Favorable	Reference				Reference			
Intermediate	1.54	0.94	2.52	0.08	1.51	0.88	2.59	0.14
Poor risk	5.56	2.98	10.38	<0.001	4.05	2.03	8.09	<0.001
**Lines of prior therapies**				0.0073				
0	Reference				Reference			
1	1.33	0.82	2.14	0.25	0.79	0.40	1.54	0.49
2	2.12	1.27	3.5	0.004	0.80	0.37	1.74	0.57
3 or more	2.10	1.22	3.62	0.008	1.60	0.73	3.54	0.24
**High NLR**	1.15	1.09	1.21	<0.001	1.12	1.05	1.19	<0.001

**Abbreviations:** ICI, immune checkpoint inhibitor; IMDC, International Metastatic RCC Database Consortium Risk model; NLR, neutrophil-to-lymphocyte ratio; PD-1, programmed cell death protein 1; RCC, renal cell carcinoma; TKI, tyrosine kinase inhibitor.

**Table 3:** Univariate and Multivariate Analysis of Factors Associated with PFS.

Predictor	Univariate				Multivariate			
	HR	95% CI		*p* Value	HR	95% CI		*p* Value
**NSAID use**	1.23	0.89	1.69	0.21	1.52	1.04	2.22	0.031
**Aspirin use**	1.18	0.86	1.61	0.30	1.39	0.95	2.04	0.087
**Male**	1.05	0.72	1.53	0.79	—			
**Age at diagnosis**	1.00	0.99	1.01	0.91	—			
**Age at treatment**	1.00	0.99	1.02	0.78	0.99	0.97	1.01	0.51
**Type of ICI**				<0.001				
PD-1 inhibitors	Reference				Reference			
PD-1 inhibitors/CTLA4 inhibitors	0.94	0.63	1.39	0.74	1.26	0.66	2.42	0.48
PD-1 inhibitors + TKI	0.76	0.40	1.46	0.42	1.16	0.56	2.42	0.69
PD-1 + interleukins under clinical trial	0.38	0.23	0.61	<0.001	0.43	0.22	0.85	0.016
**Histology subtype**				0.22				
Clear cell RCC	Reference				—			
Non-clear cell RCC	1.80	0.88	3.68	0.105	—			
**IMDC risk**				0.02				
Favorable	Reference				Reference			
Intermediate	1.11	0.77	1.62	0.57	1.25	0.82	1.9	0.31
Poor risk	2.06	1.19	3.55	0.009	1.49	0.82	2.8	0.19
**Lines of prior therapies**				0.008				
0	Reference				Reference			
1	1.62	1.10	2.39	0.014	1.28	0.7	2.33	0.42
2	1.73	1.11	2.69	0.016	1.11	0.54	2.3	0.78
3 and more	1.95	1.23	3.09	0.005	1.66	0.81	3.4	0.17
**High NLR**	1.18	1.11	1.25	<0.001	1.15	1.08	1.23	<0.001

Abbreviations: ICI, immune checkpoint inhibitor; IMDC, International Metastatic RCC Database Consortium Risk model; NLR, neutrophil-to-lymphocyte ratio; PD-1, programmed cell death protein 1; PFS, progression-free survival; RCC, renal cell carcinoma; TKI, tyrosine kinase inhibitor.

## Data Availability

The raw data supporting the conclusions of this article will be made available by the authors without undue reservation.
